# Development and Evaluation of Resident-Championed Point-of-Care Ultrasound Curriculum for Internal Medicine Residents 

**DOI:** 10.24908/pocus.v6i2.15194

**Published:** 2021-11-23

**Authors:** Leila Haghighat, Hayley Israel, Eric Jordan, Ethan L Bernstein, Merilyn Varghese, Benjamin M Cherry, Reinier Van Tonder, Shyoko Honiden, Rachel Liu, Christopher Sankey

**Affiliations:** 1 Division of Cardiology, Department of Internal Medicine, University of California San Francisco San Francisco, California USA; 2 Section of Pulmonary, Critical Care and Sleep Medicine, Department of Internal Medicine, Yale University School of Medicine New Haven, Connecticut USA; 3 Section of General Internal Medicine, Department of Internal Medicine, Yale University School of Medicine New Haven, Connecticut USA; 4 Division of Cardiology, Department of Medicine, Department of Emergency Medicine, Beth Israel Deaconess Medical Center, Harvard Medical School Boston, Massachusetts USA; 5 VA Connecticut Health System West Haven Campus West Haven, Connecticut USA; 6 Department of Emergency Medicine, Yale University School of Medicine New Haven, Connecticut USA

**Keywords:** point-of-care ultrasound, POCUS, curriculum development, resident championed, assessment

## Abstract

**Introduction: **Point-of-care ultrasound (POCUS) is a powerful clinical tool that has seen widespread adoption, including in Internal Medicine (IM), yet standardized curricula designed by trained faculty are scant. To address the demand for POCUS education at our institution, we created a resident-championed curriculum with support from skilled faculty across multiple specialties. Our objective was to teach postgraduate year (PGY)-3 IM residents the basics of POCUS for evaluation of the pulmonary, cardiac, and abdominal systems through resident-developed workshops. The goal of acquisition of these skills was for resident education and to inform decisions to pursue further patient testing. **Methods: **Three half-day workshops were created to teach residents how to obtain and interpret ultrasound images of the pulmonary, cardiac, and abdominal systems. Workshops were comprised of didactic teaching and practical ultrasound instruction with expert supervision of clinicians within and outside of IM. Residents were asked to complete a written survey before and after each workshop to assess confidence, knowledge, and likelihood of future POCUS use. **Results: **Across the three workshops (pulmonary, cardiac, and abdominal), 66 sets of pre- and post-workshop surveys (32 pulmonary, 25 cardiac, and 9 abdominal) were obtained and analyzed. Confidence in and knowledge regarding POCUS use increased significantly across all three workshops. Likelihood of future use increased in the cardiac workshop. **Conclusions: **We implemented a resident-championed POCUS curriculum that led to improved attitudes and increased knowledge of POCUS for PGY-3 IM residents.

## Background

In the last decade, advances in technology have made ultrasound technology more affordable, portable, and user-friendly. Portable ultrasound has become a valuable addition to the bedside evaluation of patients. An increasing number of clinicians across medical specialties have incorporated point-of-care ultrasound (POCUS) into practice, and professional societies have begun to codify its use. POCUS has been added to the Accreditation Council for Graduate Medical Education (ACGME) core competencies for Emergency Medicine, and the American College of Physicians (ACP) has acknowledged the important role of POCUS in Internal Medicine (IM) 1, 2. The Society of Hospital Medicine (SHM) similarly published a position statement in 2019 for Hospital Medicine specialists who use POCUS 3. 

Despite the proliferation of POCUS in clinical practice, the prevalence of formal POCUS teaching within United States IM training programs is less frequent, with only 25-31% of residencies reporting a formal curriculum 4, 5. A survey of program directors in 2018 found that fewer than half of IM residents in the US will have trained at a program with a POCUS curriculum 6. The implementation of POCUS training at the level of undergraduate medical education appears to exceed that of graduate medical education, as evidenced by a 2012 survey of US medical schools in which 62% of responding medical schools reporting having POCUS curricula 7. This discrepancy between training at the undergraduate and graduate level implies that many trainees are experiencing a decrease in POCUS education as they advance in their training, and that there is a need to increase the number of graduate programs offering curricula to make up for this difference.

In a 2010 needs assessment of medical students and IM residents, over 95% of respondents believed POCUS was useful and desired more formal training 8. Different formats and approaches have been described for building POCUS curricula within IM programs which generally emphasize the elements of having ultrasound machines, hands-on “practical” education, and didactics facilitated by dedicated POCUS faculty 9, 10, 11, 12, 13, 14. LoPresti and colleagues have also highlighted the importance of ongoing competence assessments and quality assurance 15. The principal reason cited by IM programs for the lack of POCUS education is a lack of trained instructors 16.

Resident-championed educational initiatives have been utilized for multiple facets of post-graduate medical education. The types of skills taught have included performing physical exam maneuvers, writing handoffs, and managing patients in the critical care setting 17, 18, 19. After an extensive literature search, it appears no such initiatives have been previously reported for POCUS.

We demonstrate here a resident-driven POCUS curriculum that yields measurable gains in resident POCUS education, without the need for prohibitive expert faculty time.

## Methods

### Subjects

Forty postgraduate year (PGY)-3 IM residents at Yale New Haven Hospital, which is associated with the Yale School of Medicine, during the 2018-2019 academic year were considered for this study. All but the four residents involved in the design and implementation of this study were included.

### Ultrasound Course

Residents in the Yale New Haven Hospital IM residency program rotated through the same outpatient clinic for two weeks at a time, repeated every six weeks, as part of an existing ambulatory curriculum. One half-day during each week of clinic was reserved for formal didactics, at which time our curriculum was delivered. Residents were divided into four “blocks”, which refers to the recurring two-week time period comprising a subset of residents that always rotate together during their ambulatory time. This allowed for the same two-week curriculum to be repeated for an additional six weeks to facilitate participation of all residents.

The ultrasound training was divided into three components: the pulmonary, cardiac, and abdominal systems. Curriculum content was selected to cover material that learners would not encounter elsewhere; for example, residents learn to use POCUS for procedures during Intensive Care Unit rotations, so procedural POCUS was not included. Vascular and musculoskeletal evaluation was not emphasized due to time constraints. Teaching sessions were conducted during educational half-days from October to November 2018, December 2018 to January 2019, and February to March 2019, respectively, such that each resident received instruction in each component of the course once. Every component included a one-hour didactic, followed by three hours of skill-based hands-on practice that was performed at the Yale Center for Medical Simulation or designated physical examination practice rooms at the Yale School of Medicine. Machines used for hands-on sessions included the cart-based Sparq system (Philips, Andover, MA), tablet-based Butterfly iQ (Butterfly Network, Inc., Guilford, CT), and tablet-based Lumify (Philips, Andover, MA) devices. 

Four PGY-3 IM residents with prior experience in POCUS developed the curriculum content and functioned as resident champions. Resident champion preparation consisted of completing a two-week ultrasound elective with Emergency Medicine faculty or attending a POCUS workshop at a national meeting. Expert POCUS supervisors were identified among clinicians within IM, Pulmonary/Critical Care Medicine, Cardiology, and Emergency Medicine. These faculty reviewed didactic content and taught image acquisition skills during the practical portion. A total of 18 different clinicians contributed their time and expertise, with commitments ranging from two to twenty hours per individual. For each topic component, one of the resident champions delivered the didactic portion of the session to their resident colleagues. In the skill-based portion conducted in the Yale New Haven Hospital Center for Medical Simulation and Yale School of Medicine, residents received practical experience by performing scans on one another. A subset of residents volunteered to be scanned as a model by their peers, and these residents provided verbal consent to do so. The skill-based portion started with a faculty champion demonstrating correct probe positioning and motions, after which the faculty champion would observe each resident in finding the same image, with in moment feedback to correct technique if needed. 

**Figure 1:**
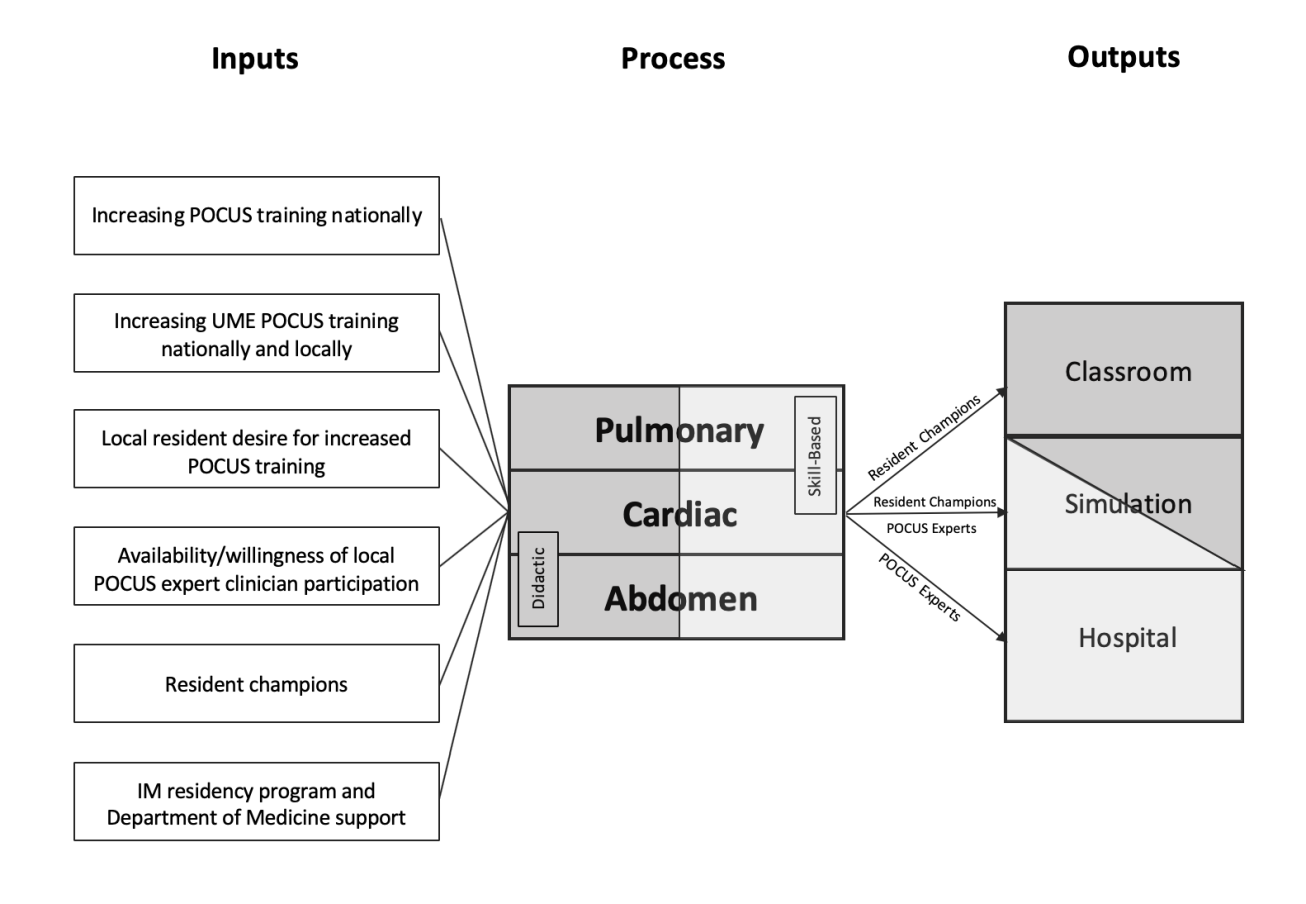
Process map of study design. Dark grey denotes those components of the curriculum that were didactic-based, and light gray denotes components of the curriculum that were skill-based.

A summary of educational content covered throughout the course is included in Table 1. A process map of the design of our POCUS course, with specific attention to where resident champions were involved, is depicted in Figure 1.

**Table 1:** Summary of educational content delivered in each of 3 POCUS sessions.

**POCUS Course** **Section**	**Abdominal**	**Cardiac**	**Pulmonary**
Educational Content	· Ultrasound physics · Probe selection · Scanning technique · Machine Interface		
	· IVC assessment · Evaluation of hydronephrosis · Evaluation of urinary retention	· Parasternal long-axis view · Parasternal short-axis view · Apical 4-chamber view · Subxiphoid view · Evaluation of pericardial effusion · Evaluation of ejection fraction · Evaluation of right-heart strain · Evaluation of aortic root dilatation	· A-lines · B-lines · Evaluation of pneumothorax · Evaluation of pleural effusion · Evaluation of pulmonary edema

### Learner Assessment 

A 10-question survey written by the resident section champion was administered before the start of the didactic (Supplementary Figure S1). The survey comprised of questions regarding confidence, knowledge, and likelihood of POCUS use in future clinical practice. Confidence and likelihood of use were assessed on a self-reported Likert scale, and knowledge was assessed using multiple-choice questions. The same survey was repeated at the conclusion of the skill-based curricular component. There was no formal assessment of the hands-on scanning component.

### Statistical Analysis 

Pre- and post-intervention surveys were compared using t-tests on Prism 8 software (GraphPad, San Diego, CA), with significance considered p<0.05.

### IRB Exemption 

The study methods and assessment instruments were submitted to the Yale University Institutional Review Board (IRB), The study was determined to be exempt from IRB review. Exemption was granted 5/23/2018 for protocol number 2000023277.

## Results

Pre- and post-test surveys were collected before and after each session. There were 32 residents present for the pulmonary sessions and 32 out of 32 (100%) completed both pre- and post- surveys. A total of 35 residents attended the cardiac sessions and 25 out of 35 (71.4%) completed both surveys. Nine total residents attended the abdominal sessions and nine out of nine (100%) completed both surveys. Attendance at the abdominal sessions was limited by scheduling conflicts.

**Figure 2:**
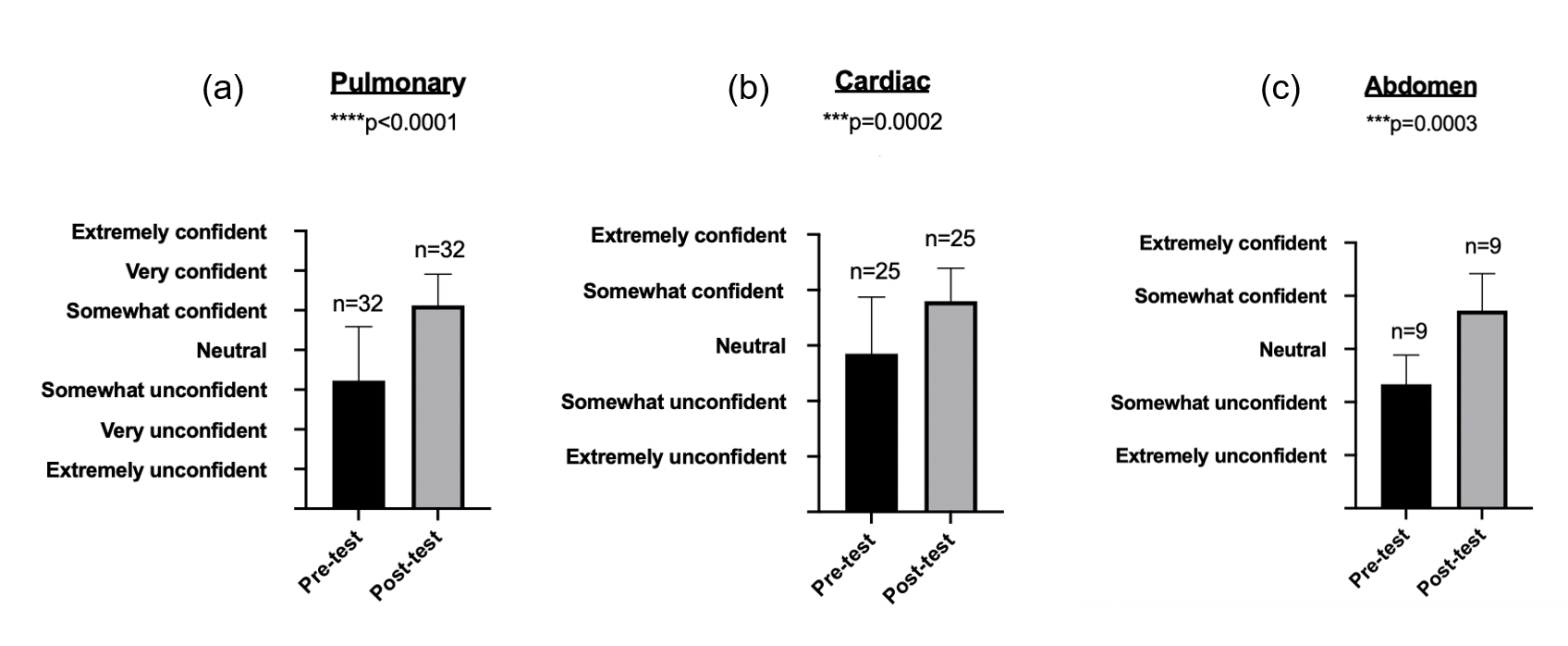
Pre- and post- assessments of resident confidence in POCUS. Confidence rose in a statistically significance manner across the cardiac (p=0.002), pulmonary (p<0.0001), and abdomen (p=0.0003) sections.

Confidence in POCUS skills improved after all sessions (Figure 2). For the pulmonary section, confidence improved from “somewhat unconfident” to “somewhat confident” (p<0.0001). For the cardiac section, residents prior to the session reported “neutral” confidence, which improved to “somewhat confident” after the session (p=0.0002). For the abdominal section, residents went from being “somewhat unconfident” prior to the session to “somewhat confident” following the session (p=0.0003).

**Figure 3:**
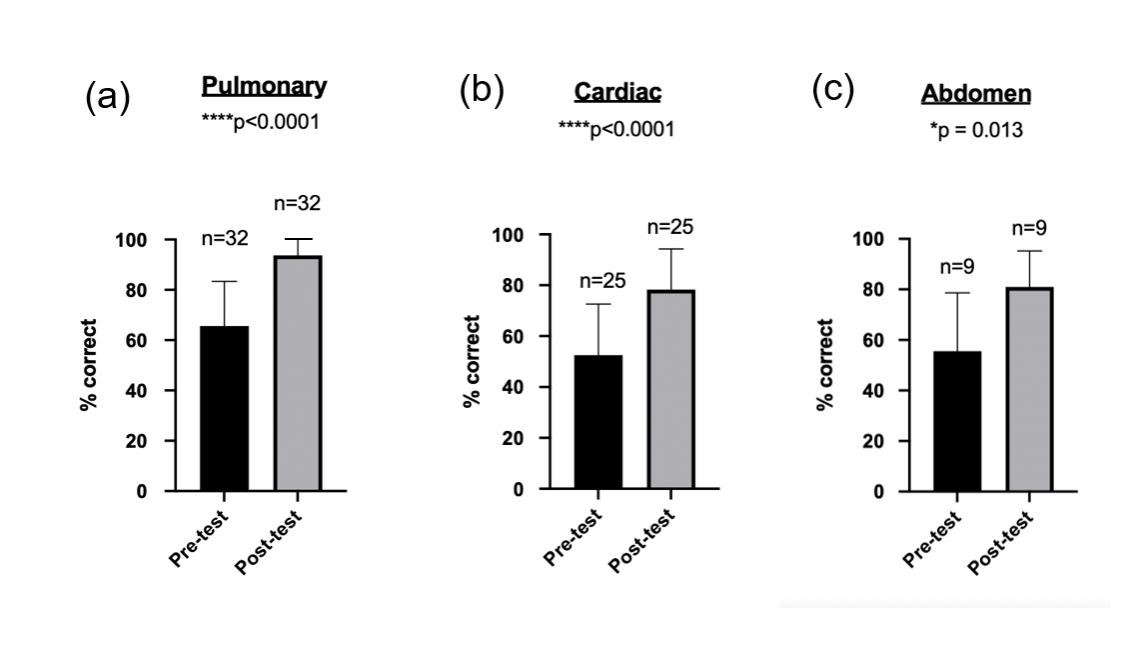
Pre- and post- assessments of accuracy on knowledge-based questions about POCUS. Accuracy rose in a statistically significance manner across all sections. (a) Mean difference before and after intervention 28.13% ± 3.56% (95% CI: 21.01% to 35.24%, p<0.0001). (b) Mean difference before and after intervention 25.71% ± 8.14% (95% CI: 15.38% to 36.05%, p<0.0001). (c) Mean difference before and after intervention 26.98% ± 11.41% (95% CI: 2.22% to 44.58%, p=0.013).

Knowledge assessment improved after all sessions, as measured by 10-question surveys (Figure 3). In the pulmonary section, average percentage correct before the session was 65.6% ± 3% compared to 93.8% ± 2% after the session (p<0.05). In the cardiac section, the average percentage correct before the session was 52.6% ± 4% compared to 78.3% ± 5% after the session (p<0.05). In the abdominal section, the average percentage correct before the session was 55.6% ± 8% compared to 81.0% ± 5% after the session (p<0.05). 

**Figure 4:**
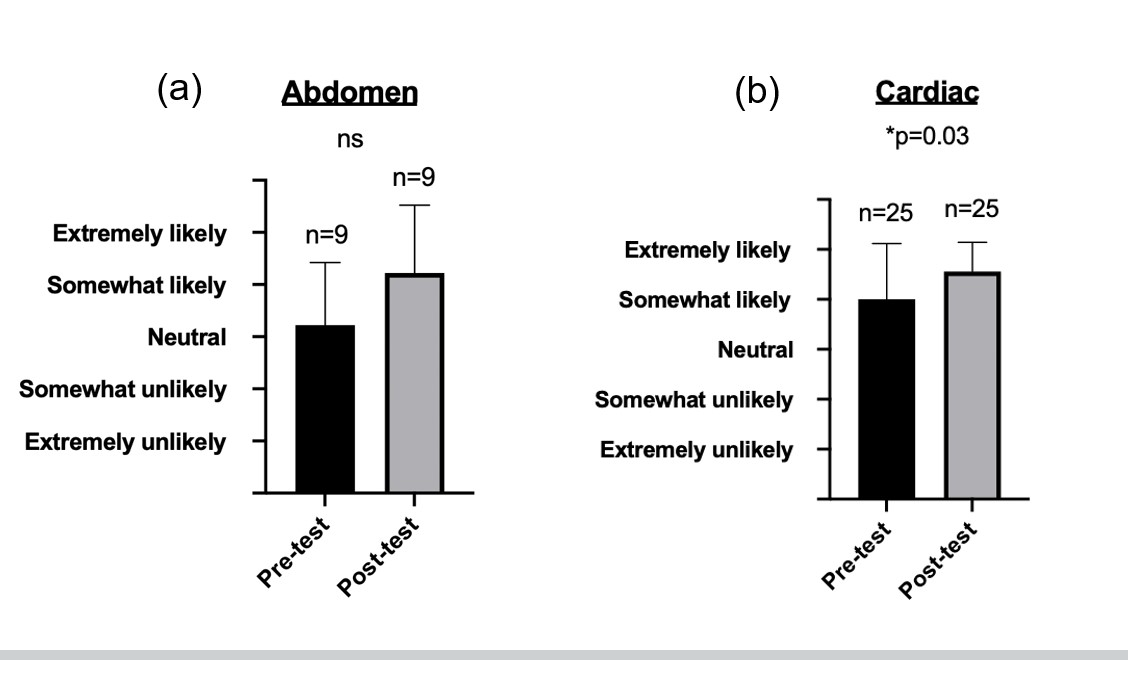
Pre- and post- assessments of resident likelihood of POCUS use. Confidence rose in a statistically significance manner in the cardiac (p=0.03) but not abdomen (p=0.11) sections.

Likelihood of use increased only in the cardiac section (Figure 4). For this section, residents were on average “somewhat unlikely” to use POCUS before the session and “extremely likely” after (p=0.03). For the abdominal section, residents went from feeling “neutral” to “somewhat likely” (p=0.11). Residents were not asked about likelihood of future POCUS use in the survey administered for the pulmonary session.

## Discussion

### Analysis

In this study, we demonstrate the feasible implementation of a resident-championed POCUS curriculum that covered the pulmonary, cardiac, and abdominal systems. The curriculum resulted in statistically significant gains in resident confidence and knowledge regarding POCUS use across all sections, with increased likelihood of use of cardiac POCUS. We believe that ultrasound abilities are best gained through a combination of formal didactics, simulation, and performing educational scans on human subjects as evidenced by the improvement residents made over a short period of time through our curriculum being set up in this manner. This may serve as a proof-of-concept for other programs who are also looking to develop resident-designed POCUS curricula.

### Strengths

One of the major strengths of this curriculum is that it was designed and implemented by resident champions, which was beneficial for engagement and participation of participants. Champions selected the scope and learning objectives for each session and created the educational content of each presentation. This peer-teaching and “bottom-up” approach to creating a longitudinal POCUS curriculum was supplemented with expert clinicians donating their time and skills in a sustainable manner. In total, 18 clinicians from multiple departments each contributed between two and twenty hours of support.

The timing of the course allowed us to reach a large number of housestaff, comprising the entire cohort of senior IM residents in our program. Additionally, the skill-based sessions resulted in interdepartmental collaboration, given that faculty and fellows from multiple specialties were present at the same time. This laid the foundation for ongoing collaborative educational efforts, such as the creation of ultrasound electives for IM residents on the hospitalist and critical care services.

### Challenges/Areas for Improvement

There are a number of limitations to this study that allow for future areas of improvement. Lack of consistency of questions on the surveys of component sections was a drawback, specifically the omission of a question asking about likelihood of future POCUS use on the pulmonary survey. Another major limitation is the lack of data from the abdominal workshops, which limits our ability to draw conclusions about one third of the curriculum. Survey completion for the cardiac sessions was only 71% which limits the generalizability of these results, however even with this limited response rate statistically significant gains in knowledge, confidence, and likelihood of future POCUS use were seen. There is additionally a need to repeat assessment of knowledge and attitudes after some time has elapsed since participation in the sessions. Lack of this information limits our ability to comment on retention of material from the workshops over time. A final constraint is lack of assessment of subsequent POCUS use for workshop participants. 

Another limitation of this study and the curriculum described is the absence of ongoing quality assurance and competence assessment, including that of hands-on scanning technique. For this curriculum to thrive, processes will need to be put in place for residents to store their images and receive ongoing feedback from POCUS faculty beyond the initial workshops. 

### Future directions

The curriculum at present only involves PGY-3s because of restraints on available time within the larger IM curriculum. Ideally, PGY-3s will teach acquired POCUS skills to junior learners in a similar fashion to how physical diagnosis skills are traditionally taught. We hope to continue to increase the time allowed for each of the component POCUS sessions within the structured educational time for residents of all PGY years. 

At present, we are beginning to evaluate our curriculum at levels one and two of the Kirkpatrick Model for evaluating training programs (levels one and two being attitude and knowledge), but our hope is that in the near future we will evaluate at level three: behavioral change 20. Level four (translation to outcomes for patient care) remains an aspirational goal at present. As residents continue to gain POCUS skills in an educational setting, rigorous quality control and assessments of competence prior to allowing residents to use POCUS for direct patient care will be necessary. Finally, as future goals we hope to demonstrate long term retention of knowledge, formal grading of image acquisition and quality, and standardized assessments through the use of observed structured clinical examinations (OSCEs).

## Conclusion

Overall, we demonstrate the feasible implementation of a resident-championed POCUS curriculum and the acquisition of improved attitudes and increased knowledge for IM PGY-3 residents.

## Conflict of Interest

All authors declare that they have no conflict of interest. 

## Supplementary Material

Supplementary Figure S1 Example of assessments used across workshops. Below is the assessment used for the pulmonary workshop. Identical assessments were used before and after each workshop to evaluate its efficacy.
