# Supplementary material for: Development and Evaluation of Resident-Championed Point-of-Care Ultrasound Curriculum for Internal Medicine Residents
Source: POCUS J. 2021 Nov 23;6(2):103–8. doi: 10.24908/pocus.v6i2.15194 (PMC9983722; doi:10.24908/pocus.v6i2.15194)
Supplement: Supplementary Figure S1 [file pocusj-06-15194-s001.pdf]

Supplements

Supplementary Figure 1. Example of assessments used across workshops. Below is the assessment used for the pulmonary workshop. Identical assessments were used before and after each workshop to evaluate its efficacy.

Bedside Ultrasound Knowledge and Attitudes Assessment

Unique Identifier: ____________________
Date: _____________________
Pre-test or Post-Test? ____________________

Do you have any prior experience with point-of-care ultrasound such as a course or any formal instruction?

1. How confident are you in using bedside ultrasound to identify a pleural effusion?

Extremely unconfident	Very unconfident	Somewhat unconfident	Neutral	Somewhat confident	Very Confident	Extremely Confident

2. How confident are you in using bedside ultrasound to identify a pneumothorax?

Extremely unconfident	Very unconfident	Somewhat unconfident	Neutral	Somewhat confident	Very Confident	Extremely Confident

3. How confident are you in using bedside ultrasound to identify pulmonary edema?

Extremely unconfident	Very unconfident	Somewhat unconfident	Neutral	Somewhat confident	Very Confident	Extremely Confident

4. Which of the following are normal findings on lung ultrasound?
   a. A lines
   b. B lines
   c. Lung Point
   d. Barcode/Stratosphere Sign

5. Which of the following is NOT a standard area to examine according to the “Blue Protocol”?
   a. Mid-clavicular line at the 2nd intercostal space
   b. Anterior axillary line, just above the nipple line
   c. Posterior axillary line at the PLAPS point
   d. Sub-scapular line, just superior to the liver

6. Which of the following is NOT an expected finding of a simple pleural effusion?
   a. Anechoic space surrounded by typical anatomic boundaries
   b. Changes in the shape of a hypoechoic area (relative to liver) with respiratory cycling
   c. Echogenic swirling pattern associated with pleural thickening
   d. Consolidated lung “floating” within the lung cavity

7. You are evaluating a 72 year-old patient with a history of HFpEF, moderate COPD, and recently diagnosed renal cell carcinoma who is presenting with the acute onset of dyspnea. You perform bedside US using the Blue Protocol and find that lung sliding is present with bilateral B lines. Which of the following diagnoses is this imaging finding most consistent with?
   a. CHF exacerbation
   b. COPD exacerbation
   c. Pulmonary Embolism
   d. Pneumothorax

8. You are evaluating a 23 year old male patient with a PMH of severe persistent asthma who presents to the ED for acute onset of dyspnea at rest. Exam is significant for tachycardia and tachypnea with normal heart and lung sounds. Notable family history includes coronary artery disease in his mother and an aortic aneurysm in his father. Bedside ultrasound shows the absence of lung sliding on the left and bilateral A lines. What is the most likely diagnosis?
   a. Asthma exacerbation
   b. Non-cardiogenic pulmonary edema
   c. Pulmonary embolism
   d. Pneumothorax

9. The following image is an example of what ultrasound finding?

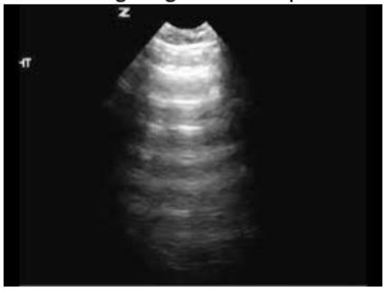

   a. A lines
   b. B lines
   c. Lung sliding
   d. Barcode/Stratosphere Sign

10. The following image is an example of what ultrasound finding?

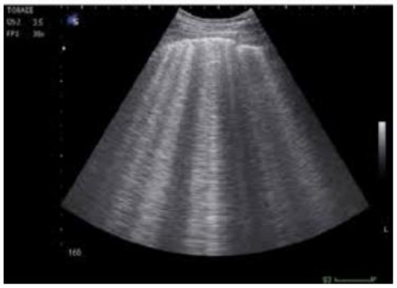

   a. A lines
   b. B lines
   c. Lung sliding
   d. Barcode/Stratosphere Sign
